# Human Serum Albumin Binds Native Insulin and Aggregable Insulin Fragments and Inhibits Their Aggregation

**DOI:** 10.3390/biom10101366

**Published:** 2020-09-25

**Authors:** Joanna Wasko, Marian Wolszczak, Zbigniew J. Kaminski, Malgorzata Steblecka, Beata Kolesinska

**Affiliations:** 1 Institute of Organic Chemistry, Faculty of Chemistry, Lodz University of Technology, Zeromskiego 116, 90-924 Lodz, Poland; joanna.wasko@dokt.p.lodz.pl (J.W.); zbigniew.kaminski@p.lodz.pl (Z.J.K.); steblecka.m@gmail.com (M.S.); 2 Institute of Applied Radiation Chemistry, Faculty of Chemistry, Lodz University of Technology, Wroblewskiego 15, 93-590 Lodz, Poland; marian.wolszczak@p.lodz.pl

**Keywords:** human insulin-labeled pyrene derivatives, spectral properties of labeled peptides, microscale thermophoresis, CD studies of complexes of HSA with insulin and insulin hot spots, aggregating properties of complexes of HSA with insulin and insulin hot spots

## Abstract

The purpose of this study was to investigate whether Human Serum Albumin (HSA) can bind native human insulin and its A13–A19 and B12–B17 fragments, which are responsible for the aggregation of the whole hormone. To label the hormone and both hot spots, so that their binding positions within the HSA could be identified, 4-(1-pyrenyl)butyric acid was used as a fluorophore. Triazine coupling reagent was used to attach the 4-(1-pyrenyl)butyric acid to the N-terminus of the peptides. When attached to the peptides, the fluorophore showed extended fluorescence lifetimes in the excited state in the presence of HSA, compared to the samples in buffer solution. We also analyzed the interactions of unlabeled native insulin and its hot spots with HSA, using circular dichroism (CD), the microscale thermophoresis technique (MST), and three independent methods recommended for aggregating peptides. The CD spectra indicated increased amounts of the α-helical secondary structure in all analyzed samples after incubation. Moreover, for each of the two unlabeled hot spots, it was possible to determine the dissociation constant in the presence of HSA, as 14.4 µM (A13–A19) and 246 nM (B12–B17). Congo Red, Thioflavin T, and microscopy assays revealed significant differences between typical amyloids formed by the native hormone or its hot-spots and the secondary structures formed by the complexes of HSA with insulin and A13–A19 and B12–B17 fragments. All results show that the tested peptide-probe conjugates and their unlabeled analogues interact with HSA, which inhibits their aggregation.

## 1. Introduction

Recently, there has been a focus on finding effective transporter vehicles for drugs. These systems, known as drug delivery systems (DDS), are characterized by properties that improve the biocompatibility, biodistribution, and physicochemical properties of a medicine once it has been administered [1]. One of the most difficult challenges associated with the design of new DDS is that they should not interact with the immune system [2,3]. This requirement is fulfilled by Human Serum Albumin (HSA), the most abundant plasma protein. This protein can reversibly bind various ligands such as lipids, hydrophobic compounds, and metal ions (copper, nickel, and calcium) with high binding affinity, acting as a carrier of various ligands in the circulatory system. It is composed of three structurally similar globular domains containing two subdomains, A and B. There are two high affinity binding sites for small heterocyclic or aromatic compounds, located on subdomains IIA and IIIA (so-called Sudlow’s site I and II, respectively), two or three long-chain fatty acid binding sites (located on subdomains IB and IIIB), and two distinct metal-binding sites, one localizing near Cys-34 and the other in the vicinity of the N terminal region [4,5,6]. However, its effectiveness and the durability of its binding properties are strongly dependent on the structure of the external ligands and on the environmental medium. The useful properties of HSA, such as biodegradability, nontoxicity, lack of immunogenicity, and the possibility for surface chemical modifications, enable the design of new complex systems able to transport biologically active compounds and treat different types of diseases [7,8,9,10]. Human Serum Albumin has emerged as a versatile carrier for therapeutic agents against cancer and infectious diseases [11,12,13,14,15,16]. Recently, fatty acid derivatives of human insulin (Levemir^®^) and Paclitaxel-HSA nanoparticles connected with clinically used antibodies (Bevacizumab, Rituximab, Trustuzumab) have been approved by the FDA as new therapeutic agents [17,18,19,20,21]. It has also been found to exhibit high affinity for the gp6 receptor, as well as binding to secreted acidic and cysteine rich protein (SPARC) and the neonatal Fc receptor (FcRn, known as a Brambell receptor or Fc fragment of IgG receptor and transporter) [22,23,24,25]. Therefore, this plasma protein has the potential to increase drug retention within tissues [26,27,28].

Reports in the literature indicate that peptides/proteins which aggregate and form insoluble deposits can be bound by HSA [29,30]. The accumulation of amyloid fibrils is a common feature of several unrelated diseases, including Alzheimer’s disease, diabetes type II, and prion diseases [31,32,33,34,35]. All of these diseases are characterized by the conversion of soluble peptide/proteins into aggregated fibrous deposits in various organs and tissues. Diabetes type II is known as a civilization disease of the 21st century. Both the number of cases and the prevalence of diabetes have been steadily increasing over the past few decades. According to the World Health Organization, about 422 million people need insulin to treat diabetes and 1.6 million deaths are directly attributed to diabetes each year. Insulin can be misfolded, forming highly ordered fibrillar amyloid aggregates both spontaneously in the body and during continuous subcutaneous insulin infusion or repeated insulin injections [36]. During the aggregation of metabolic hormones, conformational alterations in their structure may be observed, with a significant increase in the content of β-sheet-rich forms over time. Recent studies have shown that short fragments, also known as hot spots, of insulin are responsible for the initiation and acceleration of undesirable hormone aggregation in the human body [37]. The number of diseases caused by the presence of insoluble amyloid deposits in organs and tissues has inspired the search for new methods of diagnosis and visualization, as well as more effective treatments [38]. In the case of insulin, it is important to understand the mode of hormone transport in the organism and to find conditions that may decrease the tendency for amyloid formation without interfering with the biological activity of the hormone. On the other hand, in addition to the problem of insulin aggregation, biological activity should be ensured in the case of treatment with insulin or its derivatives. It was shown that HSA conjugates with insulin formed by using the thiol group on Cys34 HSA had longer half-lives compared to the native hormone [39]. HSA plays the role of both a transporter and a system protecting peptide pharmaceuticals against the action of proteolytic enzymes and conformation changes, which improve the pharmacokinetic (PK) properties of drugs [40]. The positive effect of the protective role of HSA on the extension of half-lives of polypeptides is particularly evident in the case of lipidated derivatives of insulin, GLP-1 analogs, and hGH [41], which is related to numerous HSA domains that effectively bind hydrophobic compounds. Moreover, albumin fusion technology has been used to obtain both long-acting insulin analogues [42] and HSA-fusion products with Tregitope peptides and insulin peptides useful in the treatment of Type 1 Diabetes [43].

In studies at the cellular level, a useful technique is to apply fluorescent labeled molecules to compounds [44]. Techniques based on the application of fluorescence dye probes have many advantages, such as fast detection, good repetitiveness, and low required dosages [45,46]. A simple measurement of fluorescence intensity can be qualitatively and/or quantitatively correlated with the presence of the labeled object either inside or outside cells. It also enables conformational changes within biomolecules and biological membranes to be followed, while facilitating early diagnosis of diseases and enabling the biodistribution of drugs to be controlled [47,48].

Pyrene and its derivatives are very popular fluorescent markers used to study interactions with HSA [49,50,51,52]. Despite the fact that there is differentiated localization of pyrene derivatives in HSA, these systems are commonly applied as fluorescent probes, due to the high sensitivity of their fine vibronic structure to changes in the environment (the Ham effect) and long fluorescence lifetimes. The chromophore itself binds with albumins in a nonspecific way, with relatively low affinity (binding constant in the order of 10^4^ dm^3^ mol^−1^), and its polarity index (*I*_1_*/I*_3_ ratio of vibronic peaks in the fluorescence spectrum) is close to that in ethanol. Negatively-charged pyrene derivatives also bind strongly within albumins [52]. In a study of the intermolecular proton-transfer reaction of tri-sodium 8-hydroxypyrene-1,3,6-trisulfonate (pyranine, HPTS) with HSA, Cohen et al. suggested that the probe is bound in Sudlow’s site II [53]. There are also data in the literature concerning the design of pyrenyl probes attached to short peptides chains, which are capable of recognizing specific sites in proteins (e.g., in lysozyme and bovine serum albumin) [54,55,56] and the specific structural elements of cell membranes. These systems, also known as protein scissors activated by light, are responsible both for recognition of the binding site in the target molecule and for producing the photochemical energy necessary to selectively destroy peptide bonds inside the protein. Furthermore, derivatives of 4-(1-pyrenyl)butyric acid can be used to determine oxygen concentrations in biological systems [57,58].

The data on the ability of HSA to bind various compounds prompted us to undertake research on the use of HSA as a system capable of transporting peptidic inhibitors of insulin and amylin aggregation (both of these hormones are present in the amyloid deposits identified in people with diabetes). Before starting research with the use of peptidic inhibitors of the aggregation process, it was necessary to ascertain whether HSA interacts (binds) with native hormones as well as their fragments known as hot spots. Hot spots are structural elements that determine the aggregation of native polypeptides (proteins). The first goal of the project was therefore to determine whether HSA binds native human insulin and the A13–A19 and B12–B17 fragments of human insulin, which are responsible for aggregation of the whole hormone. We assumed that attaching 4-(1-pyrenyl)butyric acid as a fluorophore to insulin and both hot spots of insulin would enable localization of their binding sites within HSA because of its spectral properties. The use of pyrene derivative of butyric acid should enable the synthesis of fluorophore labeled peptides directly on solid support or giving the opportunity of attachment it to insulin under conditions assuring a preservation of the unchanged spatial structure of the hormone. Additionally, we expected that the binding of insulin and its hot spots by HSA would influence their susceptibility to aggregation. The impact of HSA could thereby be tested in this respect. We next investigated whether HSA interacted with insulin and its fragments, and whether HSA reduced the aggregation capacity of insulin and its hot spots. Confirmation of the ability of HSA to bind insulin and its fragments and reduce their susceptibility to aggregation might then provide the basis for further research on the use of HSA as DDS for peptidic inhibitors of the insulin aggregation process, which could lead to innovative approaches to diabetes treatment.

## 2. Materials and Methods

### 2.1. General Information

Human recombinant insulin (Sigma-Aldrich, Poznan, Poland), essentially fatty acid free human serum albumin (HSA) (Sigma-Aldrich), 4-(1-pyrenyl)butyric acid (Merck, Warsaw, Poland), human recombinant insulin (Sigma-Aldrich), Congo Red, Thioflavin T (Sigma-Aldrich, Poznan, Poland), and all necessary amino acid derivatives (Merck) were used as received. NaH_2_PO_4_∙2H_2_O, Na_2_HPO_4_∙12H_2_O, TX-100, EtOH, MeOH, and NaCl (Sigma-Aldrich) were used for the buffer preparation. Water was purified with the use of a Millipore Milli-Q Plus system.

Analytical HPLC: UltiMate 3000 UHPLC System Thermo Scientific™; column parameters: Kinetex 2.6 u C18 100A, 100 × 4.6 mm, 20 °C; diode array UV/Vis detector (DAD); eluent ACN/H_2_O; gradient 0–2 min 3/97, 2–31 min 95/5, 31–32 min 0/100, 32–33 min 0/100, 33–35 min 3/97, 35–37.5 min 3/97.

Preparative HPLC: CombiFlash, EZPrep, Teledyne ISCO, Supelco Discovery BIO Wide Pore C18 column (25 cm × 21.2 mm, 10 mm; Sigma Aldrich); flow rate, 5 mL/min; detection wavelengths, 220 and 254 nm); gradient ratio A (0.1% TFA in ACN)/ B (0.1% TFA in H_2_O) 0:100 to 18:82 in 30 min, followed by an isocratic run for 5 min.

ESI/MS: micrOTOF-Q III spectrometer Bruker Daltonics equipped with electrospray source (ESI) and time of flight detector (TOF).

### 2.2. Synthesis of Py-Ins (**1**) Conjugate

To a vigorously stirred and cooled to 5 °C solution of 4-(4,6-dimethoxy-1,3,5-triazin-2-yl)-4-methyl-morpholinium *p*-toluenosulphonate (DMT/NMM/TosO^−^) [59] (37.2 mg; 0.09 mmol) in DMF (2 mL) was added 4-(1-pyrene)butyric acid (26 mg; 0.09 mmol) and N-methylmorpholine (NMM) (7.42 μL, 0.068 mmol). The progress of activation was controlled by TLC (eluent: DCM, visualization: NBP solution). After consumption of the whole amount DMT/NMM/TosO^−^ (following the disappearance of the colored spot with R_f_ = 0 and the appearance of spots with R_f_ = 0.3), the solution was added to human insulin (174 mg, 0.03 mmol) dissolved in phosphate buffer pH 8 (5 mL) and 3 equiv. of NMM (9.9 μL, 0.09 mmol) were added. The reaction was continued for 24 h. Anal. RP-HPLC: R_t_ = 18.3 min; purity of crude product = 45%. LC/MS: 1622.9230 ([M+4H]^4+^; C_297_H_411_N_65_O_79_S_6_^4+^; calc. 6348.198).

### 2.3. Synthesis of Py-LYQLENY (**2**) and Py-VEALYL (**3**)

Peptides H-LYQLENY-OH (**5**) (A13–A19) and H-VEALYL-OH (**6**) (B12–B17) were synthesized in a Liberty Blue Automated Microwave Peptide Synthesizer (CEM Corporation, Matthews, NC, USA) on chlorotrityl resin according to the Fmoc/tBu protecting group strategy. Functionalization of the obtained peptides by 4-(1-pyrene)butyric acid was carried out in a syringe reactor using DMT/NMM/TosO^−^ as a coupling reagent.

#### 2.3.1. Loading of the 2-Chlorotrityl Chloride Resin (GP1)

The amino acid (3 equiv. relative to the resin) and 6 equiv. of EtN*i*Pr_2_ were dissolved in CH_2_Cl_2_ (10 mL/g resin). The 2-chlorotrityl chloride resin was pre-swollen in CH_2_Cl_2_ for 1 h. The solution of amino acid and the resin were added and the suspension was shaken for 120 min. The resin was then filtered and washed with CH_2_Cl_2_/MeOH/EtN*i*Pr_2_ in the ratio of 17:2:1 (3×), DMF (2×) and CH_2_Cl_2_ (3×). The functionalized resin was dried in a vacuum desiccator to constant mass.

#### 2.3.2. Standard Coupling Procedure (GP2)

Peptide synthesis was performed at 0.1 M scale in an Automated Microwave Peptide Synthesizer. All reagents were dissolved in DMF. In a vessel, 1 equiv. of the appropriate amino acid (0.2 M), 1.5 equiv. of the coupling reagent (DMT/NMM/TosO^−^, 0.5 M), and 4.8 equiv. of NMM (2 M) were added automatically to the resin. The Fmoc protecting group was removed using 20% piperidine in DMF. For each amino acid, one cycle of coupling included: initial deprotection (time, *t* = 30 s; temperature, *T* = 75 °C; microwave energy, *P* = 25 W), deprotection (*t* = 180 s; *T* = 75 °C; *P* = 35 W), coupling (*t* = 300 s; *T* = 75 °C; *P* = 22 W). 

#### 2.3.3. Peptides Functionalization by 4-(1-Pyrene)butyric Acid (GP3)

To the obtained peptides on resin was attached 4-(1-pyrene)butyric acid as a fluorescent probe. Before coupling, the resin was pre-swollen in CH_2_Cl_2_ for 1 h. Activation of the carboxyl group of 4-(1-pyrene) butyric acid was performed in DMF. First, 2 equiv. of DMT/NMM/TosO^−^ was dissolved and stirred for 5 min at 0 °C. Then, 2 equiv. of fluorescent probe and 1 equiv. of NMM were added. The progress of the reaction was controlled using TLC (eluent CH_2_Cl_2_, visualization: UV-Vis and 0.1% NBP solution in EtOH). The lack of a red spot and R_f_ = 0 indicated the end of the activation process. The solution was transferred to swollen resin without a Fmoc group on the N-terminal amino acid. Coupling was performed for 24 h. The resin was filtered and washed with DMF (3×) and CH_2_Cl_2_ (3×). Completion of the reaction was monitored by the Kaiser test.

#### 2.3.4. Cleavage from the Resin (GP4)

The conjugates functionalized with fluorescent probe were cleaved from the resin using a mixture of 95% TFA (2,2,2-trifluoroacetic acid)/2.5% H_2_O/2.5% TIS (triisopropylsilane), (2 mL/0.1 g resin). Cleavage was performed over 4 h, and then the resin was filtered off and the filtrate was evaporated under nitrogen. To the residue was added Et_2_O to precipitate the product. The obtained solid was filtered off and washed with Et_2_O. The crude product was lyophilized and identified by the LC-MS method. 

#### 2.3.5. Synthesis of Py-LYQLENY-OH (**2**) Derived from Fragment A13-A19 of Human Insulin

In an Automated Microwave Peptide Synthesizer, 0.145 g of resin (0.1 mmol) was esterified with Fmoc-Tyr(tBu)-OH, according to GP1. Subsequently, the peptide chain was elongated with: Fmoc-Asn(Trt)-OH (0.72 g), Fmoc-Glu(OtBu)-OH (0.52 g), Fmoc-Leu-OH (0.78 g), Fmoc-Gln(Trt)-OH (0.74 g), Fmoc-Tyr(tBu)-OH (0.56 g), and Fmoc-Leu-OH (0.39 g) in the presence of DMT/NMM/TosO^−^ (3.1 g) and NMM (8 mL) according to GP2. The synthetized peptide was functionalized by 4-(1-pyrene)butyric acid in accordance with GP3: DMT/NMM/TosO^−^ (82 mg, 0.2 mmol), 4-(1-pyrene)butyric acid (57 mg, 0.2 mmol), NMM (11 μL, 0.1 mmol). The final product was cleaved from the resin (GP4). Anal. RP-HPLC: R_t_ = 22.5 min; purity of crude product = 77%. LC/MS: 1212.5231 ([M+H]^+^, C_64_H_77_N_9_O_15_^+^; calc. 1212.328).

#### 2.3.6. Synthesis of Py-VEALYL-OH (**3**) Derived from Fragment B12-B17 of Human Insulin

In an Automated Microwave Peptide Synthesizer, 0.127 g of resin (0.1 mmol) was esterified with Fmoc-Leu-OH, according to GP1. Subsequently, the peptide chain was elongated with: Fmoc-Tyr(tBu)-OH (0.41 g), Fmoc-Leu-OH (0.43 g), Fmoc-Ala-OH (0.38 g), Fmoc-Glu(OtBu)-OH (0.52 g), Fmoc-Val-OH (0.41 g) in the presence of DMT/NMM/TosO^−^ (3.1 g), and NMM (8 mL) according to GP2. The peptide was functionalized by 4-(1-pyrene)butyric acid in accordance with GP3: DMT/NMM/TosO^−^ (82 mg, 0.2 mmol), 4-(1-pyrene)butyric acid (57 mg, 0.2 mmol), NMM (11 μL, 0.1 mmol). The final product was cleaved from the resin (GP4). Anal. RP-HPLC: R_t_ = 25.4 min; purity of crude product = 78%. LC/MS: 977.4645 ([M+H]^+^, C_54_H_68_N_6_O_11_^+^; calc. 977.132).

#### 2.3.7. Synthesis of H-LYQLENY-OH (**5**) (A13–A19), H-VEALYL-OH (**6**) (B12–B17)

Peptides **5** and **6** were used for research [60].

### 2.4. Preparation of Samples for Measurements 

#### 2.4.1. Absorption and Fluorescence Measurements of Py-Ins, Py-LYQLENY, Py-VEALYL

The solutions of HSA (concentration = 3 10^−5^ M) were prepared by dissolving the protein in 0.01 M of phosphate buffer saline (PBS, [NaCl] = 150 mM), pH = 7.2. Due to the partial aggregation of Py-Ins (**1**), Py-LYQLENY (**2**), and Py-VEALYL (**3**), we were not able to determine the molar absorbance coefficients of these compounds in the buffer solution. The examined samples were obtained by dissolving small amounts of Py-Ins (**1**), Py-LYQLENY (**2**) and Py-VEALYL (**3**) in HSA solution and vortexing for less than 1 min. The prepared samples were used within 24 h. Py-Ins (**1**), Py-LYQLENY (**2**), and Py-VEALYL (**3**) were also dissolved in Triton TX^®^ solution (TX-100), methanol, and ethanol. The concentration of HSA was verified spectrophotometrically using the molar absorption coefficient 35500 M^−1^cm^−1^ at 280 nm for HSA [61]. In order to ensure complete protein hydration, the solutions were stored at room temperature for at least 1 h before measurements. The samples for fluorescence measurements were saturated with argon or deaerated with a vacuum pump. The results were independent (within experimental uncertainty) of the deaeration procedure.

#### 2.4.2. MST Measurements 

HSA was used as a fluorescently labeled target molecule. Peptides **5** and **6** (ligands) without a fluorescent marker were used in the studies. The HSA (0.6 mg) was stained using RED-NHS second-generation dye, diluted in dimethyl sulfoxide. The remaining solutions and subsequent ligand dilutions were prepared in 1 × PBS buffer with the addition of 0.1% Pluronic^®^ F-127 solution and sodium chloride (150 mM). The final concentration of HSA used in experiments was 20 nM, whereas the highest concentration for both ligands was 0.25 mM. A serial 1:1 dilution was performed by transferring one volume of ligand solution to an equal volume of buffer, mixing, and repeating. The ligand concentration was reduced by 50% in each dilution step. After serial dilution of the ligand, 10 µL of labeled HSA was added to each tube from 16 to 1 and mixed by pipetting. After 30 min of incubation at ambient temperature, the capillaries were dipped into the appropriate tube with solution and then put in the device tray.

#### 2.4.3. CD Measurements

Samples were prepared by dissolving 1 mg of the analyzed peptide in phosphate buffer pH 7.2 (10 mL), to a final concentration of 0.1 mg/mL All measurements were carried out at room temperature using a quartz cuvette (1 mm path length, Hellma). The experimental settings were as follows: measurements range, 190–270 nm; data pitch, 5 nm; scanning mode, continuous; scanning speed, 100 nm/min; bandwidth, 3 nm; integration time, 1 s.

#### 2.4.4. UV-Vis Measurements in the Presence of Congo Red

Insulin, peptides **5** and **6**, and HSA (final concentration 2.88 mM), as well as each sample of the mixture of insulin, peptides **5** and **6**) (1.44 mM) with HSA (1.44 mM), were incubated in phosphate buffer solution at pH 7.2 (1 mL) for 7 days at a temperature of 37.2 °C. Then, 1 mL of a solution of Congo Red dye (45 μM in phosphate buffer, pH 7.2) was added to each incubated sample. Incubation continued for another 3 days. During this time, spectroscopic measurements of all the samples were performed in the range of 400–800 nm. The UV-Vis spectra recorded on the first, second, and third days of incubation for all samples are shown in the Supplementary Material (Figures S1 and S2).

#### 2.4.5. Intensity of Fluorescence Measurements in the Presence of Thioflavin T

Insulin, peptides **5** and **6,** and HSA (final concentration 0.66 mM), as well as each sample of the mixture of insulin, peptides **5** and **6**) (0.33 mM) with HSA (0.33 mM) were incubated in phosphate buffer solution, pH 6.0 (2 mL), for 7 days at a temperature of 37.2 °C. Then, 2 mL of a solution of Thioflavin T (57 mM in phosphate buffer, pH 6.0) was added to the samples. Incubation continued for another 3 days. During this time, spectroscopic measurements of all samples were performed in the range of 470–600 nm, excitation wavelength 440 nm. The intensity of fluorescence spectra recorded on the first, second, and third days of incubation for all samples. are shown in the Supplementary Material (Figures S3 and S4).

#### 2.4.6. Microscopic Measurements

Microscopic studies were carried out for samples prepared in phosphate buffer (0.1 M, pH 7.2) incubated for 7 days at 37.4 °C. To 2 mL of H-LYQLENY-OH (**5**) and H-VEALYL-OH (**6**) (c = 2.88 mM) solution was added 2 mL of HSA solution (c = 0.60 mM). The final concentration of incubated peptides was equal to c = 1.44 mM, whereas, for plasma protein, it was 0.30 mM. Incubation was also performed for 4 mL of an HSA sample (c = 0.3 mM). After that period, 4 mL of CR solution (c = 45 μM in phosphate buffer, pH 7.2) was added to the all samples, which were incubated for another 3 days at room temperature.

### 2.5. Measurement Methods

Steady-state absorption and fluorescence spectra were recorded at a resolution of 0.5 nm using an Aminco Bowman AB2 Series spectrofluorometer (Thermo Fisher Scientific Inc., Waltham, MA, USA). Unless otherwise indicated, in emission measurements, the excitation wavelength was 337 nm, and excitation and emission slits were set to 2.0 and 0.5 nm, respectively.

Time-resolved measurements of HSA fluorescence were performed using a flash photolysis system based on a GL-3300 nitrogen laser (Photon Technology International, Birmingham, NJ, USA) providing single light pulses with a wavelength of 337.1 nm, duration ~800 ps, and average energy 100 µJ. The detection system consisted of a Baush&Lomb monochromator (Rochester, NY, USA), Hamamatsu 1P28 photomultiplier, and a PS325 power supply system (Stanford Research, Sunnyvale, CA, USA). Output signals from the photomultiplier were digitized and recorded using a Tektronix DPO 7254 oscilloscope (Beaverton, OR, USA) (40 GS/s) and transferred to a computer. All experiments were carried out at room temperature (25 ± 1 °C) using quartz cells with an optical length of 1 cm.

The CD spectra were recorded using a Jasco J-1500 instrument (Jasco, Cracow, Poland) and quartz cuvettes with 1 mm optical path lengths. The measurements were acquired in the range of 190–270 nm at ambient temperature by taking points every 5 nm, with a scan rate of 100 nm per min, an integration time of 1 s, and a bandwidth of 4 nm. The samples were prepared in phosphate buffer (0.1 M, pH 7.2) with a final concentration of 0.1 mg/mL.

The MST measurements were carried out using a Monolith NT.115 (NanoTemper Technologies GmbH, München, Germany) instrument in special Monolith NT.115 capillaries.

The microscopic assays were performed for samples stained with Congo Red dye. The samples were transferred on a microscopic slide and visualized using a Delta Optical Genetic Pro (Warsaw, Poland) light microscope.

The UV-Vis measurements were carried out using a Hitachi spectrophotometer (Hitachi, Tokyo, Japan).

The fluorescence intensity measurements were carried out using a FLUOROMAX-4 system from Horiba Scientific (Edison, NJ, USA), measurement range of 470–600 nm, excitation wavelength 440 nm.

## 3. Results and Discussion

**Figure 1:**
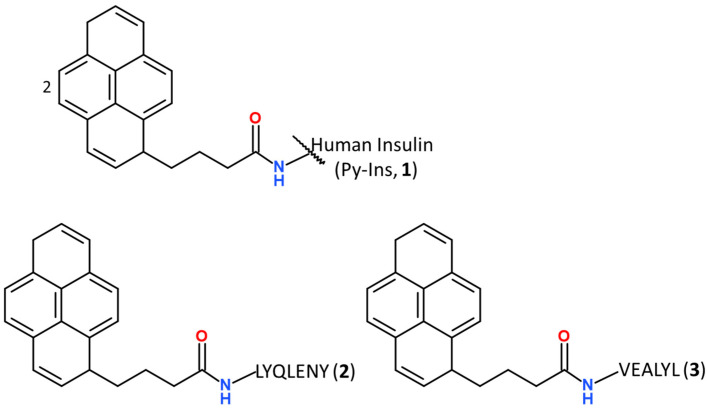
The structure of labeled insulin (**1**) and insulin amyloidogenic cores (**2**, **3**) with 4-(1-pyrene)butyric acid.

A derivative of insulin labeled with 4-(1-pyrene)butyric acid Py-Ins (**1**) was used for the tests, as well as two labeled insulin amyloidogenic cores: Py-LYQLENY-OH (**2**) analog of A13-A19 and Py-VEALYL-OH (**3**) derived from the B12-B17 fragment (Figure 1). Py-Ins conjugate (**1**) was obtained by coupling human insulin with 4-(1-pyrene)butyric acid, using 4-(4,6-dimethoxy-1,3,5-triazin-2-yl)-4-methyl-morpholinium *p*-toluenosulphonate (DMT/NMM/TosO^−^) [59] as a coupling reagent. The synthesis of derivatives (**2**) and (**3**) was performed under microwave-assisted standard solid phase peptide synthesis conditions, also in the presence of DMT/NMM/TosO^−^ as a coupling reagent. This strategy enabled pure products to be obtained with high efficiency and avoided undesirable side reactions, such as racemization and folding of peptide chains. Conjugates **1**–**3** with at least 98% purity were used in the studies.

The first stage of the research concerned the determination of the spectral characteristics of the HSA–4-(1-pyrene)butyric acid (PBA) complex. 4-(1-pyrene)butyric acid was used as a negative control in studies of the interaction of HSA with derivatives **1**–**3**. We have analyzed the interaction of PBA with HSA in some detail (Figure S5). The observed changes of fluorescence intensity are complex. Addition of HSA to aqueous PBA solution the intensity (I) decreased, reaching a minimum at the PBA/HSA molar ratio 2, and then increased to some extent. A similar trend was also observed for the complex of 1-pyrene sulfonic acid (PSA) with HSA [52]. The intensity (I) decreasing can be due to the interaction with quencher Trp214 residue located in the domain IIA. However, the quenching efficiency of PBA decreased at HSA concentrations higher than 8 μM. This finding can suggest that PBA may also be located in another site (sites) distant from Trp214 (non-specific binding). The decay of the excited singlet state of PBA (15 μM) in a wide range of HSA concentrations for deaerated samples are shown in Figure S6. Additionally, it has been found that, in the case of a sample of PBA in buffer (without of HSA), fluorescence decay can be described as a classical first-order kinetics, and its lifetime is equal to 126 ns (Table S1). The biexponential kinetic patterns and the lifetime data as a function of the HSA concentration (Table S1) might be interpreted in terms of different microenvironments of the probe. The fast component, *τ_1_* (in the range of 16–20 ns), most likely corresponds to PBA molecules embedded in the subdomain IIA (near Trp214) [52]. The higher HSA concentrations led to successively docking of the probe to the protein; *τ_2_* increases monotonically reaching the limit value 163 ns. The important observation from time-resolved measurements is the change in the primary fluorescence signal intensity (Figure S6) in the function of HSA concentration. The literature data show that PBA itself has a weak affinity for albumin [56,62]. 

**Figure 2:**
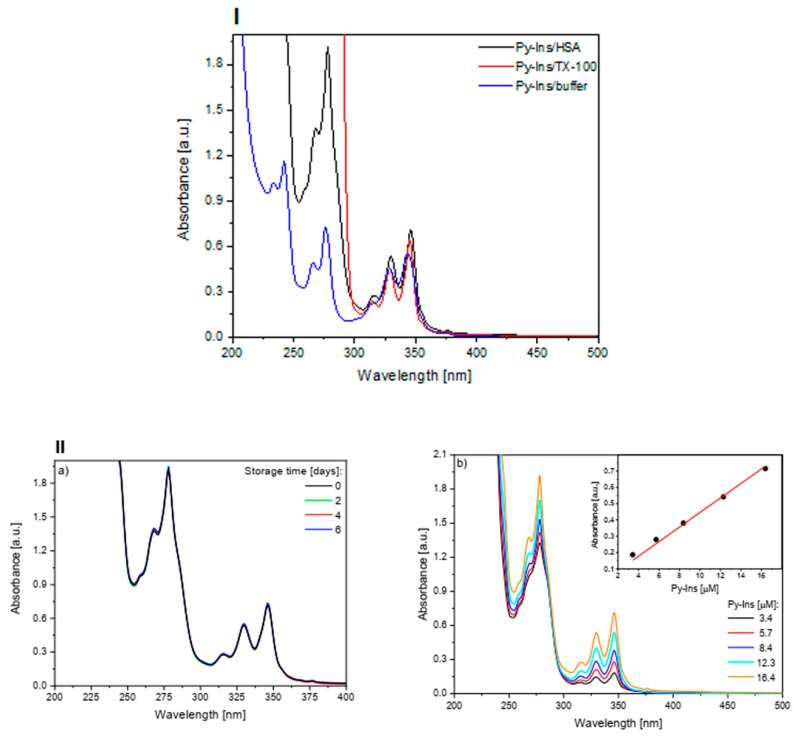
Panel I: Absorption spectra of Py-Ins (**1**) in PBS, and with additives: TX-100 or HSA. [Py-Ins] = 16.38 μM, [TX-100] = 20 mM, [HSA] = 30 μM. Panel IIa: Absorption spectra of HSA/Py-Ins (**1**) complex, [Py-Ins] = 16.38 μM, [HSA] = 30 μM recorded for the fresh solution and after 2, 4, and 6 days of storage. Panel IIb: Absorption spectra of HSA/Py-Ins (1), [HSA] = 30 μM, [Py-Ins] as indicated (inset: dependence of A342 on the Py-Ins concentration).

We began our research by determining the spectral characteristics of the Py-Ins conjugate in the phosphate buffer (PBS), with HSA or TX-100 as additives (conditioning the formation of micellar structures) (Figure 2, panel I). In the buffer solution, the maximum of the band corresponding to the S_0_ (ν = 0) → S_2_ (ν = 2) transition of the pyrene moiety occurred at 342 nm. The addition of 30 μM HSA or 20 mM TX-100 resulted in characteristic red-shifts of 2 and 3 nm, respectively, and an increase in absorbance with characteristic better separation of the vibrational structure. The observed spectral changes are characteristic for the transfer of the fluorescent marker to the more hydrophobic environment. 

Broad absorption bands can indicate the presence of Py-Ins aggregates. Therefore, a solution of Py-Ins (**1**) complex with HSA was filtered through a 0.2 µm syringe filter in order to remove any Py-Ins aggregates, and the stability of the complex was tested for a further 6 days. On the absorption spectra recorded after 2, 4, and 6 days (Figure 2, panel IIa), no changes were observed, illustrating the stability of the complex and a lack of Py-Ins aggregation. This may indicate that the Py-Ins (**1**) conjugate was completely bound to the HSA, and that the protein has a protective role in the aggregation process. Additional confirmation of this was provided by the observed linear increase in Py-Ins absorbance for the samples diluted with 30 µM HSA solution (Figure 2, panel IIb).

**Figure 3:**
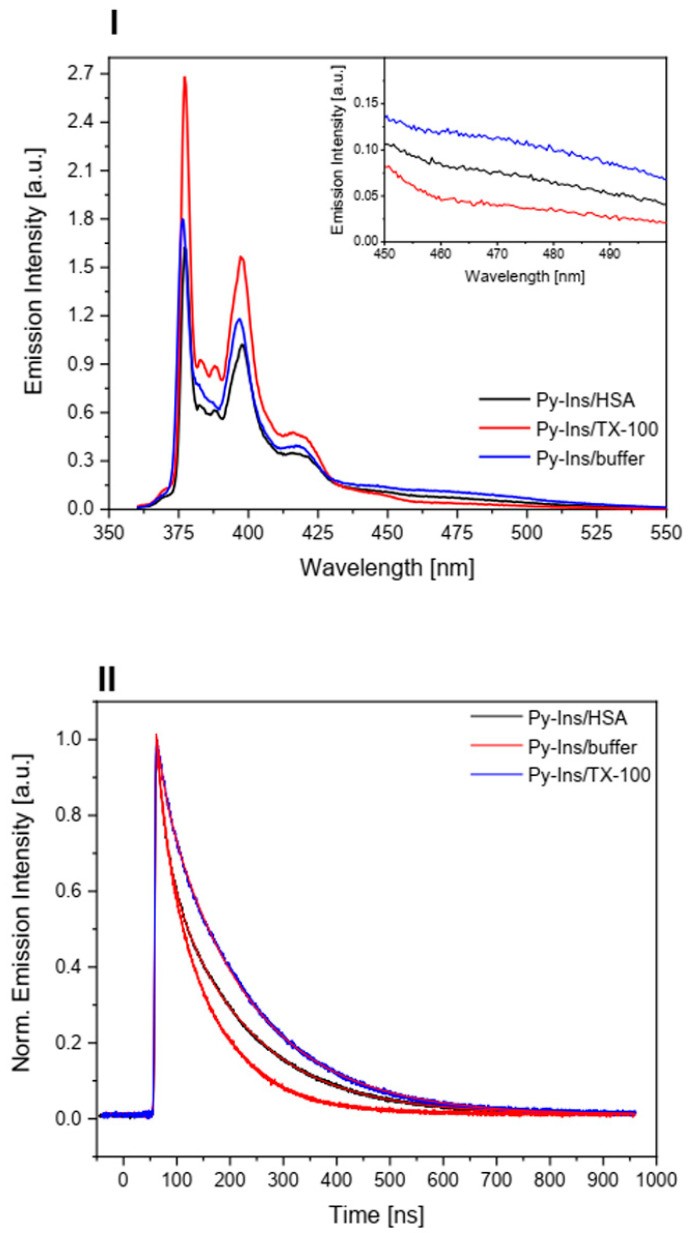
Panel I: Fluorescence spectra of Py-Ins (**1**) in aqueous buffer solution and in the presence of TX-100 or HSA. [Py-Ins] = 16.38 μM, [TX-100] = 20 mM, [HSA] = 30 μM, λ_exc_ = 337 nm. Inset: the same spectra in the range of 450–500 nm. Panel II: Normalized decays of Py-Ins (**1**) fluorescence in PBS, TX-100, and HSA. [Py-Ins] = 16.38 µM, [TX-100] = 20 mM, [HSA] = 30 μM, λ_exc_ = 337 nm, λ_em_ = 405 nm. See Table 1 for parameters of the best-fit lines (smooth).

The binding capacity of the Py-Ins (**1**) conjugate by HSA was also demonstrated by the results of fluorescence studies (Figure 3, panel I). Measurements were made in PBS and with the addition of TX-100 or HSA. Typically, the transfer of a pyrene marker from an aqueous environment to a less polar environment leads to higher emission intensity and longer lifetime in the excited state, due to the lower impact of nonradiative mechanisms for energy dissipation in the hydrophobic environment. This was confirmed in the case of Py-Ins (**1**) in TX-100 solution. The different behavior of Py-Ins in the presence of HSA suggests more effective fluorescence quenching of the pyrene molecule docked to the HSA binding site. In the presence of TX-100 and HSA, the pyrene fluorescence spectra show a prominent fine structure in the form of five predominant peaks, while, on the spectra for the pyrene derivatives in buffer solution, this vibrational structure was not detected in full. The lack of vibronic peaks *I*_2_ and *I*_3_ on the fluorescence spectrum of Py-Ins (**1**) may point to the presence of aggregates of the Py-Ins conjugate or a different microenvironment. The spectra presented in Figure 3, panel I show a characteristic broad band in the spectral range of 450–500 nm, which may be associated with the formation of excimers of pyrene fragment bounded to insulin or exciplexes between pyrene residue and an aromatic amino acid of the HSA.

In homogeneous buffer solutions, the Py-Ins (**1**) fluorescence decay can be described by the bi-exponential function:G

(1) $$\left(t\right)=I_{0}+A_{1}\exp\left(-\frac{\left(t-t_{0}\right)}{\tau_{1}}\right)+A_{2}\exp\left(-\frac{\left(t-t_{0}\right)}{\tau_{2}}\right)\;$$

where *A_1_*, *A_2_* are amplitudes of the lifetime components *τ_1_*, *τ_2_*.

We applied a 4-parameter fitting procedure and the best fit results are summarized in Table 1, together with the average lifetimes calculated as:

(2) $$<\tau >\;=\frac{\sum^{\;}A_{i}\tau_{i}^{2}}{\sum^{\;}A_{i}\tau_{i}}\;$$

The fast component (*τ_1_*, *A_1_*) and the slow component (*τ_2_*, *A_2_)* can be assigned to Py-Ins (**1**) molecules in the aggregate and monomer forms, respectively. Transfer of the probe from the buffer solution to the TX-100 matrix or HSA was accompanied by an increase in the mean lifetime of the singlet excited state from 86.6 ns to 147.9 ns and 133.6 ns, respectively. Filtering of the solution also increased the fluorescence lifetime for all the examined systems. The data presented in Table 1 indicate that the *A_1_* amplitude reflecting the contribution of the aggregate fraction decreased significantly after filtering the solution and for Py-Ins/TX-100 the kinetic decay became nearly monoexponential. However, even for diluted solutions, Py-Ins/HSA ([Py-Ins]/[HSA] = 0.11 (data not shown), the aggregate fraction made up about 20%. The results obtained for Py-Ins (**1**) derivative show that pyrene chromophore attached to insulin penetrate deeper area inside HSA, whereas the peptidic tale of insulin can stabilize the HSA-Py-Ins (**1**) complex by interaction of the peptide with the HSA surface. Moreover, the superficial locus of the hormone does not dramatically change the environment inside the HSA.

**Figure 4:**
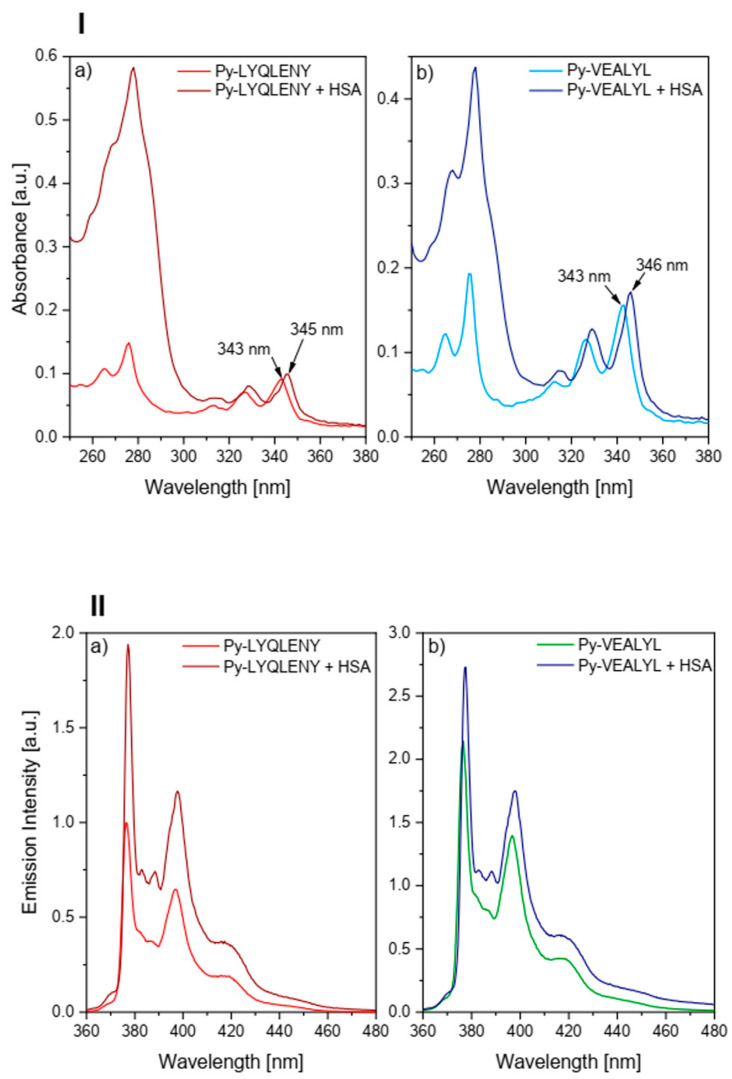
Panel I: absorbance spectra of (**a**) Py-LYQLENY (**2**) and (**b**) Py-VEALYL (**3**) in the presence and absence of HSA. [HSA] = 30 μM. Panel II: fluorescence spectra of (**a**) Py-LYQLENY and (**b**) Py-VEALYL in the presence and absence of HSA. [HSA] = 30 μM, λ_exc_ = 337 nm.

The results of our research on the ability of HSA to bind the conjugate Py-Ins (**1**) prompted us to undertake further studies using labeled 4-(1-pyrene)butyric acid of amyloidogenic insulin cores: Py-LYQLENY-OH (**2**) and Py-VEALYL-OH (**3**). In order to investigate the binding of Py-LYQLENY (**2**) and Py-VEALYL (**3**) to HSA, absorption studies as well as steady-state and time-resolved fluorescence studies were performed, without and in the presence of the serum protein, using PBS solution s of **2** and **3**. The spectral properties of peptides **2** and **3** are presented in the Supplementary Material (Figures S7 and S9). The absorption spectra of both pyrene-labeled insulin fragments are presented in Figure 4, panel I), in which a 2–3 nm bathochromic shift in the presence of HSA compared to the buffer solution may be observed. This finding indicates the preferred location of the pyrene label within the less polar area within HSA. Moreover, peptidic fragments could interact with the HSA surface, enhancing docking of the probe inside the protein. Similar observations for pyrene-short synthetic peptide conjugates have been presented by Kumar et al. [54,55,56]. 

Figure 4 (Panel II) shows the emission spectra of pyrene-labeled peptides (**2**) and (**3**) in the presence and absence of HSA. In the presence of HSA, there was a significant change in the oscillation structure compared to the PBS solutions, involving the appearance of previously invisible vibrational bands II and III in the pyrene moiety. This confirms the interaction of the pyrene-labeled insulin fragments with HSA, and the change of the microenvironment of the fluorescence probe from strongly polar (buffer solution) to hydrophobic (less polar) inside of HSA. In the presence of HSA, the pyrene moiety ceases to be exposed to a polar medium and starts to be located in a hydrophobic microenvironment. Stiffening of its structure probably took place (the carbon atoms of the pyrene residue are situated in the same plane), which accounts for the sharpening of the oscillating structure in the spectrum. 

**Figure 5:**
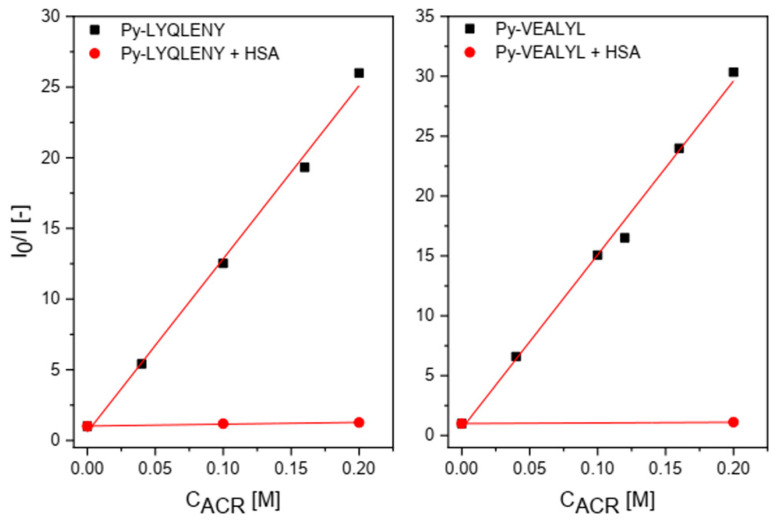
Stern–Volmer plots for quenching of Py-LYQLENY and Py-VEALYL by ACR in the buffer and HSA solution. I_0_ is fluorescence intensity without quencher, I—fluorescence intensity at various concentrations of ACR, [HSA] = 30 µM.

Additionally, we examined the quenching of Py-LYQLENY and Py-VEALYL by acrylamide (ACR) in the buffer solution and HSA solution (Figure 5 Stern-Volmer plots). In the presence of HSA, both conjugates **2** and **3** were protected against interaction with the quencher, since the slope of the relationship *I*_0_/*I* vs. ACR concentration is much lower with respect to the analogous slope in the case of the protein. These results indicate that pyrene fragment of both conjugates Py-LYQLENY (**2**) and Py-VEALYL (**3**) is located inside of HSA. 

**Figure 6:**
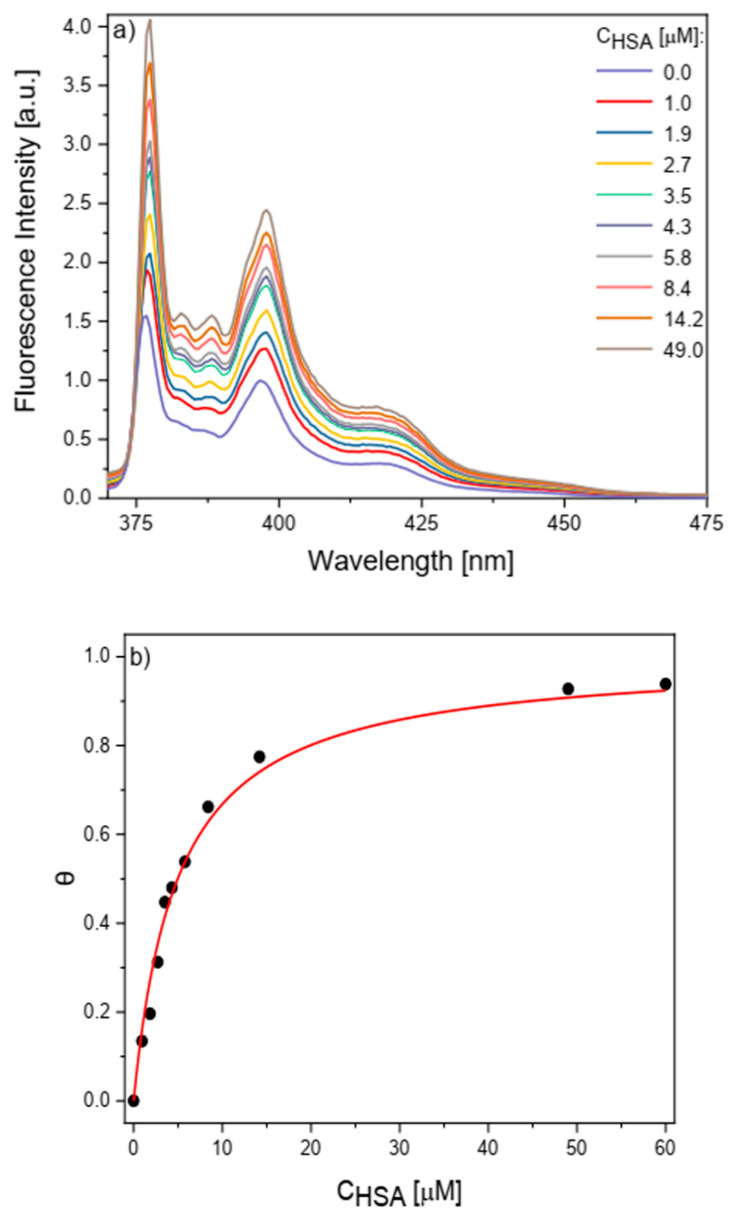
(**a**) fluorescence spectra of Py-LYQLENY (3 µM) with HSA in buffer solution; (**b**) dependence of parameter θ on HSA concentration.

For Py-LYQLENY conjugate (**2**), the binding constants K_b_ were calculated according to Equation (3) proposed by Kuzmin et al. [63]:

θ = K_b_·[HSA] / (1+ K_b_·[HSA]) (3)

where [HSA] is the concentration of the albumin [61], θ = (I − I_0_)/(I_∞_ − I_0_) is the fraction of the pyrene probe bound with HSA, I_0_ is fluorescence intensity without HSA, I_∞_ is fluorescence intensity with [HSA] = 45 µM (reaching a plateau, Figure 6b) and I is the fluorescence intensity for subsequent acrylamide (ACR) concentrations used as a fluorescence quencher. 

Based on measurements of fluorescence intensity as a function of HSA concentration (Figure 6a), it was found that K_b_ of Py-LYQLENY (**2**) equals 2 × 10^5^ dm^3^ mol^−1^. The calculated value also confirmed existence of interactions between 2 and HSA with pyrene fragment presented on less polar environment inside protein. It has been proved that the trapping of PSA inside HSA led to two possible probe/protein binding modes with the binding constants 6.5 × 10^6^ dm^3^ mol^−1^ (a specific receptor site), and 3.8 × 10^5^ dm^3^ mol^−1^ (non-specific binding) [52]. The binding constants 1.5 ± 0.3 × 10^6^ dm^3^ mol^−1^ to 6.5 ± 0.4 × 10^7^ dm^3^ mol^−1^ were calculated for complexes of HSA with Py-hydrophobic peptide. Attachment of 4-(1-pyrene)butyric acid to peptide chain containing Phe and Gly residues can increase the hydrophobicity of docked molecules and improve the more specific binding [56]. 

**Figure 7:**
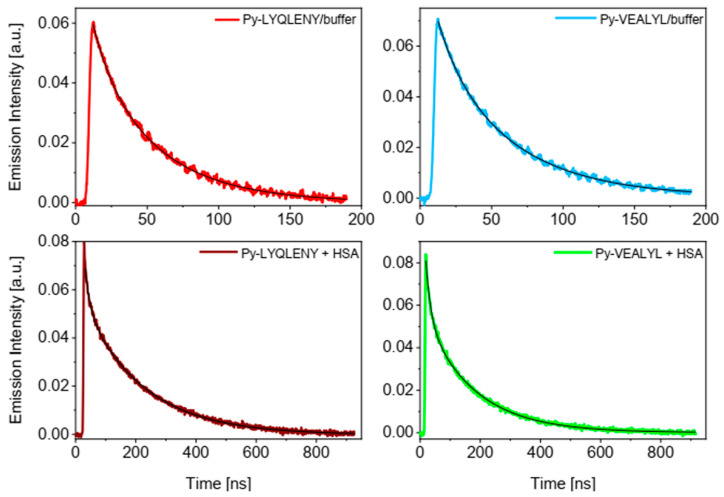
Time profiles of Py-LYQLENY (**2**) and Py-VEALYL (**3**) fluorescence decay in buffer and in the presence of HSA. Smooth lines represent best-fits to experimental curves. [HSA] = 30 μM, λ_exc_ = 337 nm, λ_em_ = 400 nm.

The results of steady state fluorescence studies were also confirmed by time-resolved measurements of fluorescence decay for the fluorescence marker both in the buffer and in the presence of HSA (Figure 7). In buffer, the fluorescence of the probe decayed according to classical first-order kinetics, and its lifetime *τ_0_* was determined by fitting the experimental decay with the exponential function. For HSA conjugates **2** and **3**, a bi-exponential function was applied and the average lifetimes were calculated from Equation (2). The times of the fluorescence decay for conjugates **2** and **3** are summarized in Table 2. 

**Figure 8:**
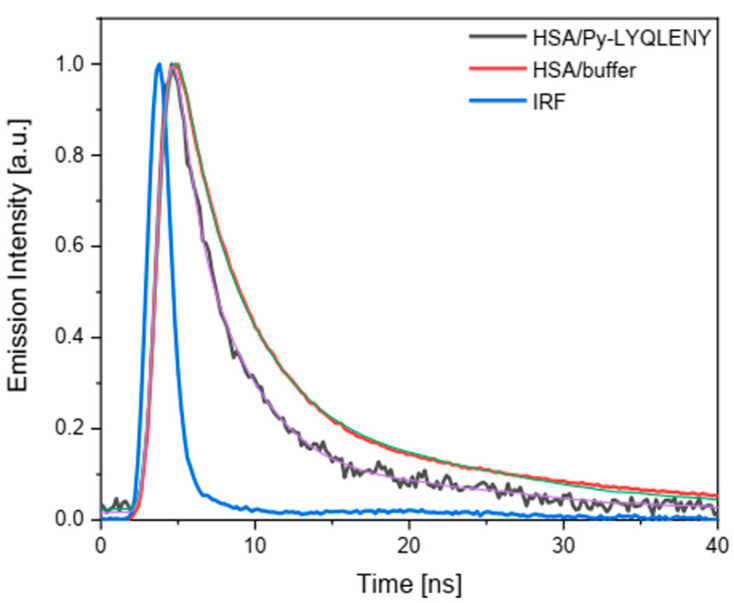
Decay curves of excited Trp214 in the presence of Py-LYQLENY; [HSA] = 30 µM, λ_exc_ = 295 nm, λ_em_ = 340 nm. Smooth lines: bi-exponential fits to experimental runs.

Our results show that the fluorescence lifetimes of both conjugates were about four times longer in the presence of HSA compared to the PBS solution. The remarkable prolongation of pyrene fluorescence lifetime in both cases confirmed preferred probe localization in a less polar environment inside protein. To examine possible interaction with Trp214, we employed the time-resolved technique with excitation at 295 nm to track the fluorescence changes of Trp214 in subdomain IIA (Sudlow’s I site). The results presented in Figure 8 reveal a slight decrease in the Trp214 fluorescence lifetime in the presence of Py-LYQLENY. 

The decay curves were fitted using the bi-exponential function and the average lifetimes were 5.4 ns and 3.7 ns for HSA/PBS and HSA/Py-LYQLENY, respectively. HSA fluorescence (*Trp214) was quenched by Py-LYQLENY via Förster resonance energy transfer (Figure 8). The parameters of FRET analysis for HSA/Py-LYQLENY (including r_DA_ = 22 Å) fall in the range of reported data [52]. This can be evidence of Py-LYQLENY specific binding in the Sudlow’s site 1. Additionally, we noticed another confirmation of this thesis in the pulse radiolysis measurements of the HSA—Py-LYQLENY. complex. It has been claimed [64] that ligands located at the Sudlow’s site 1 are not reduced by the hydrated electron. We found that Py-LYQLENY (**2**) is efficiently reduced by hydrated electron in a buffer solution even in concentration as low as 20 µM (Figure S10), while, in the presence of HSA, the reduction process of Py-LYQLENY has been completely eliminated.

Based on the results presented so far, it can be concluded that HSA shows the ability to transport and inhibit undesirable aggregation of Py-Ins, Py-LYQLENY, and Py-VEALYL conjugates. In order to confirm the results of spectroscopic measurements, we also analyzed the interactions of native insulin (**4**) and hot spots of insulin (LYQLENY (**5**), VEALYL (**6**)), using three independent research techniques covering circular dichroism, microscale thermophoresis techniques, and a set of nonspecific assays recommended for the study of protein/peptide aggregation (the Congo Red (CR) assay, Thioflavin T (ThT) assay, and microscopic examination).

**Figure 9:**
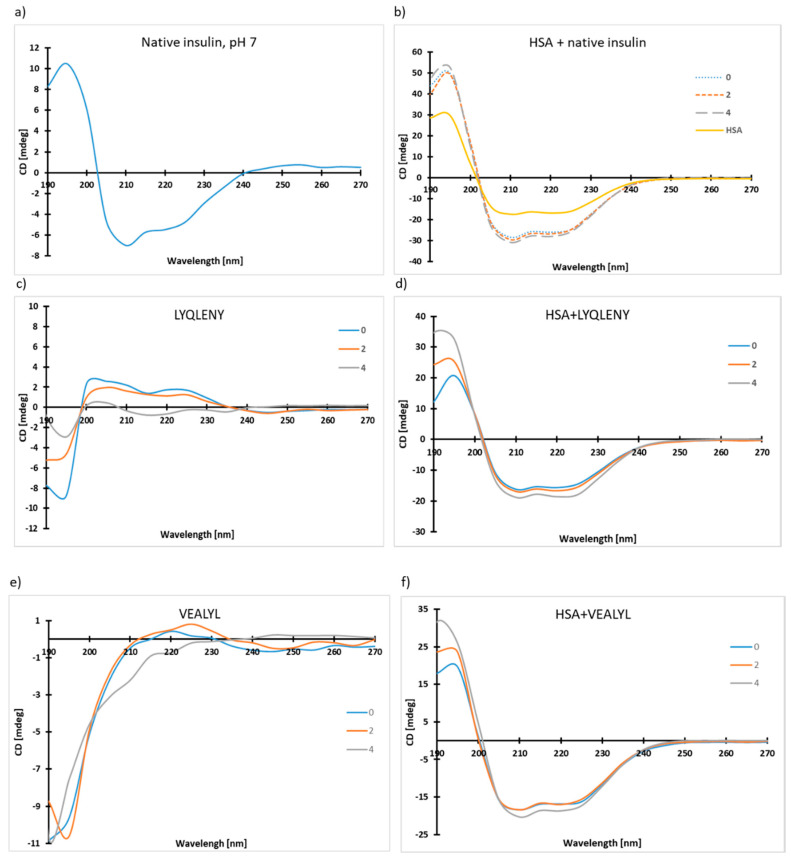
CD spectra recorded for native insulin (**4**) and hot spot fragments (**5**–**6**) in the presence of HSA and without serum protein: (**a**) native insulin, (**b**) HSA-insulin complex, (**c**) H-LYQLENY-OH (**5**), (**d**) HSA- LYQLENY complex, (**e**) H-VEALYL-OH (**6**), (**f**) HSA-VEALYL complex. [HSA], [insulin] [LYQLENY], [VEALYL] = 0.1 mg/mL.

Circular dichroism (CD) spectroscopy can be a very valuable source of information about differences in the secondary structure of peptides and proteins [65]. It has been shown that all α-helical proteins show a strong minimum at 222 nm and 208–210 nm and a strong maximum at 191–193 nm. In the case of β-sheet protein, a single negative band may be observed in the 210–225 nm wavelength range and a strong positive band in the 190–200 nm wavelength range, although their intensities are noticeably lower than those for α-proteins [66]. The CD spectra recorded for buffered solutions of insulin (**4**) and hot-spot fragments (**5**), (**6**) in the presence of HSA as well as for native insulin are presented in Figure 9a,b. The measurements were performed after 30 min (sample 0), and then after two and four days of incubation at 37 °C.

On the CD spectra of the HSA-insulin complex (Figure 9), a regular increase was observed in signal intensity at 194 nm and a stronger minimum at 210 and 224 nm. This means that, in the presence, of the native form of insulin does not tend to form amyloid fibrils, for which the β-sheet structure is the main growth factor [60,67]. We can suppose that possible interactions between HSA and the native hormone inhibit undesirable insulin aggregation and increase the content of the α-helix structure in the sample. Changes recorded for the HSA-LYQLENY complex (Figure 9c,d) were slightly different compared to the HSA-insulin complex. Most importantly, no systematic increase was noticed in the intensities of the maximum and minimum. After four days of incubation, the signal intensity reached the highest volume (195, 210, and 223 nm), which also confirmed the increase in the amount of the α-helix structure as a result of binding of LYQLENY by HSA. On the other hand, the signal intensity at 223 nm showed the same enhancement as the signal at 210 nm. The observed increase of the minimum at 223 nm indicates the existence of a small quantity of misfolded LYQLENY. Moreover, this fraction showed a tendency to form β-sheet and/or loop structures in buffer solution, resulting in a significantly signal increment at 223 nm. There was also a lack of regular changes in signal intensity at 193 nm observed for the HSA-VEALYL complex (Figure 9e,f), although for this hot spot the increase in the intensity of the minimum at 210 nm was more pronounced in comparison to that of the minimum at 224 nm. All changes found on the CD spectra for the HSA-VEALYL complex confirmed that this hot spot also interacts with HSA. Binding of VEALYL by HSA prevents the formation of amyloid fibrils. Additionally, we can suppose that the non-bound fraction of VEALYL in the presence of HSA has less tendency to misfold than LYQLENY, as evidenced by the smaller content of β-sheet structures in the buffer solution.

**Figure 10:**
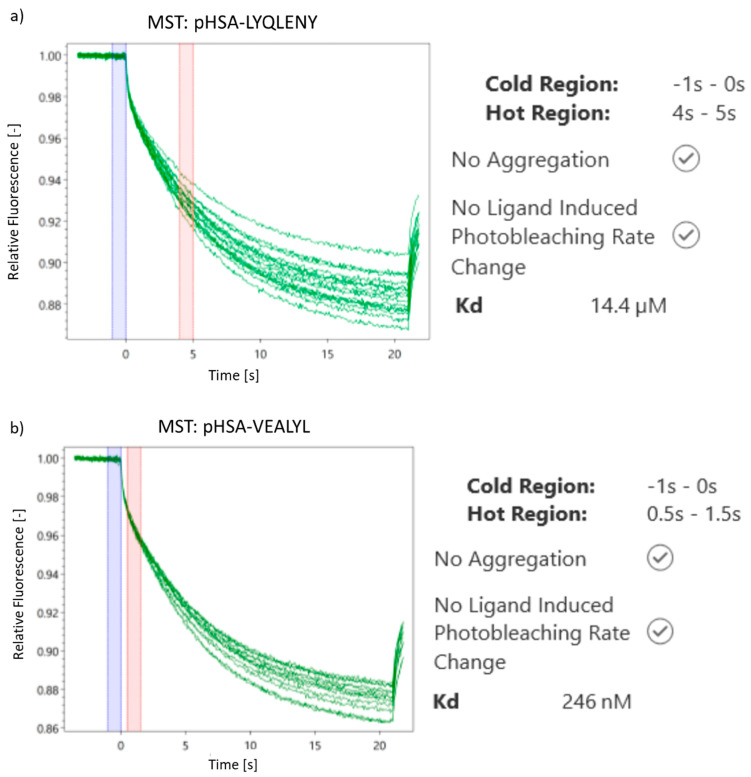
MST spectra recorded for: (**a**) complex of H-LYQLEN-OH (**5**) with HSA, (**b**) H-VEALYL-OH (**6**) with HSA.

The second technique used to assess the inhibition of aggregation of insulin (**4**) and insulin hot spots **5** and **6** in the presence of HSA was the microscale thermophoresis (MST) assay. This assay [68,69] is a very powerful and practical technique for quantifying interactions between small molecules and protein fibrillar aggregates. For both complexes of HSA with **5** and **6**, it was possible to estimate the dissociation constant (K_d_), which confirmed the attachment of the external ligand to the labeled protein. For LYQLENY (Figure 10a), K_d_ equals 14.4 µM, whereas, for the VEALYL fragment (Figure 10b), K_d_ equals 246 nM. The results indicate stronger interactions for the HSA-VEALYL complex, which is consistent with the results obtained from CD measurements. Moreover, the regular fluorescence changes observed with increasing concentrations of a non-fluorescent ligand show the limited tendency of both hot spots to aggregate in the presence of HSA.

**Figure 11:**
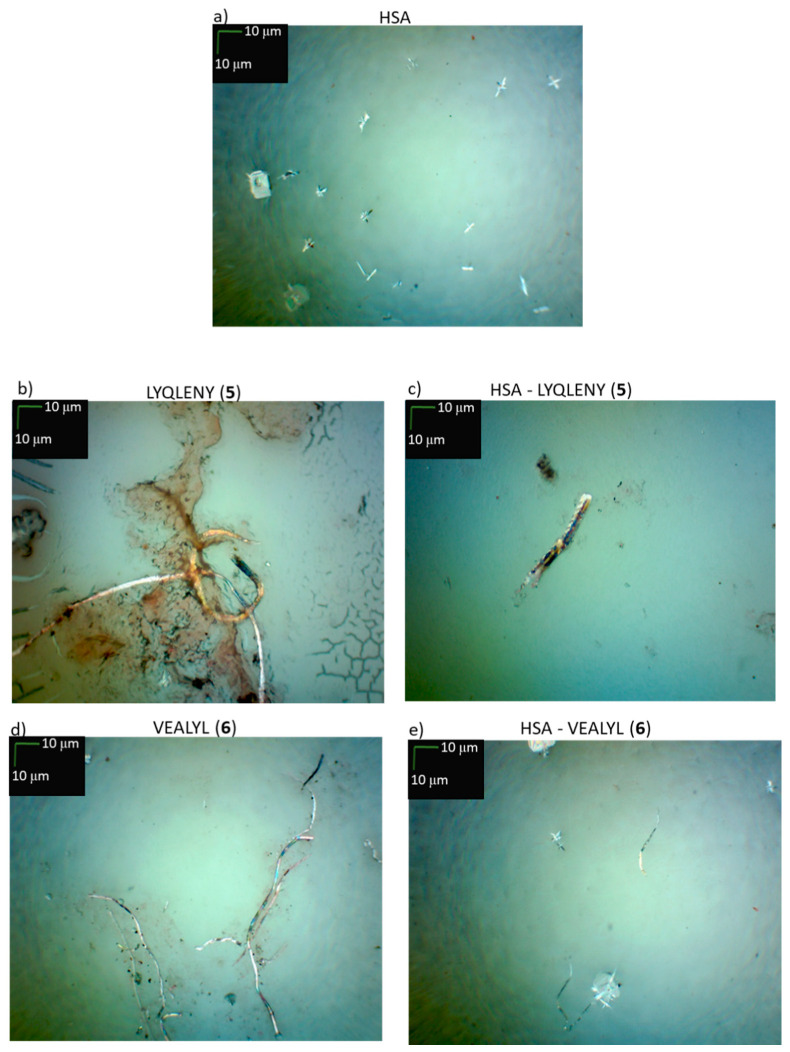

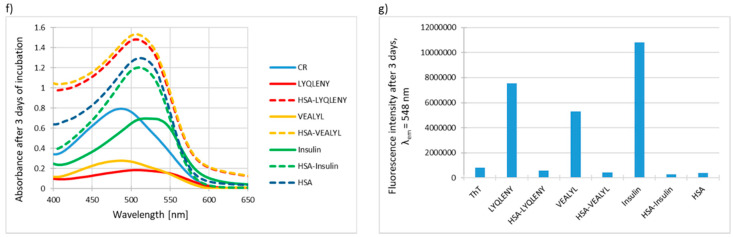
The results of studies on the susceptibility of complexes of HSA with insulin and its hot spots **5** and **6** to aggregation. Microscopic examination for: (**a**) HSA, (**b**) H-LYQLENY-OH (5), (**c**) HSA-LYQLENY, (**d**) H-VEALYL-OH (6), (**e**) HSA-VEALYL. Scale bars, 10 μm, microscopic measurements were performed on the third day of incubation. (**f**) UV-Vis spectra of insulin, peptides **5** and **6**, HSA, complexes of HSA with insulin, and peptides **5** and **6** in the presence of Congo Red (CR). (**g**) Intensity of fluorescence of insulin, peptides **5** and **6**, HSA, complexes of HSA with insulin and peptides **5** and **6** in the presence of Thioflavin T (ThT), after the third day of incubation.

In the last stage of our study, we tested the effect of HSA on the aggregation capacity of insulin and its hot spots. Three independent research techniques recommended for non-specific testing of peptide/protein aggregates were used. One of the methods was based on the examination by microscopy of peptide aggregates stained with CR. In the sample containing only HSA (Figure 11a), we recorded a crystal structure characteristic for proteins. Hot spot fragments **5** and **6** after incubation at 37 °C formed typical amyloid fibers (Figure 11b,d), which were very clearly visible after staining with CR [60]. In the presence of HSA, we observed significantly less content of fibers characteristic for amyloid deposits (Figure 11c,e). Moreover, the observed structures have different sizes and shapes and were less efficiently stained by CR in comparison to fibers formed by H-LYQLENY-OH and H-VEALYL-OH in the absence of HSA. Thus, the results of microscopy assays also proved that HSA can bind hot spots fragments, simultaneously enhancing their solubility and limiting the formation of amyloid deposits in buffer solution.

In the CR assay, no decrease in absorbance typical for amyloid structures was observed in the case of any HSA complexes with insulin and hot spots **5** and **6** (Figure 11f). A shift in the absorption maximum was observed, which might suggest the formation of amyloid aggregates. However, we found that this is the typical course of the UV-Vis curve for HSA. 

The results of the ThT assay also without cross-talk showed the absence of amyloid aggregates of HSA complexes with insulin and peptides **5** and **6** (Figure 11g). In all cases, we found no increase in fluorescence intensity, which was comparable to the values obtained for ThT.

## 4. Conclusions

In this study, we labeled insulin and its hot spots **5** and **6** with 4-(1-pyrene)butyric acid using DMT/NMM/TosO^−^ as a coupling reagent. Both in a solution and in solid phase, this strategy was found to very effective, allowing products to be obtained with the desired spectroscopic properties. For the derivatives Py-Ins (**1**), Py-LYQLENY (**2**), and Py-VEALYL (**3**), labeled with the pyrene marker, fluorescence spectra showed a decay of the pyrene probe oscillation structure. We also observed a shortening of the fluorescence lifetime for Py-Ins (**1**), Py-LYQLENY (**2**), and Py-VEALYL (**3**) in PBS to 86.6 ns, 38.3 ns, and 43.9 ns, respectively. These results indicate peptide aggregation. However, in the presence of HSA, the solubility of Py-Ins (**1**), Py-LYQLENY (**2**), and Py-VEALYL (**3**) increased significantly in aqua media. There was also a noticeable change in the oscillation structure of the fluorescence spectra compared to those for samples without the serum protein. Prolongation of the fluorescence lifetimes was also observed, to 133.6 ns, 186.2 ns, and 153.9 ns for Py-Ins (**1**), Py-LYQLENY (**2**), and Py-VEALYL (**3**), respectively. All of these data indicate the inhibition of aggregation by derivatives **1**, **2**, and **3** in the presence of HSA. Moreover, for all labeled conjugates, the pyrene chromophore was able to penetrate deeper within the HSA. In turn, the peptidic fragment could interact with HSA surface moieties, stabilizing probe docking. The slight reduction of fluorescence lifetime of tryptophan residue Trp-214 (located in Sudlow’s I site) in the presence of Py-LYQLENY (**2**) proves possible localization of pyrene core in this less polar area. However, the possibility of docking pyrene labeled peptides in different hydrophobic HSA pockets cannot be ruled out. Pyrenyl modified peptides bind to HSA simultaneously in a specific and non-specific manner with similar binding constant of 10^5^ dm^3^ mol^−1^.

The results for unlabeled native insulin (**4**) and its hot spot fragments H-LYQLENY-OH (**5**) and H-VEALYL-OH (**6**) were consistent with data from spectroscopic measurements. Circular dichroism (CD) studies indicated increased amounts of the α-helical structure in all analyzed samples after incubation. It can be supposed that, in the presence of HSA, native insulin (**4**) as well as hot spot fragments (**5**) and (**6**) have less tendency to misfold, as evidenced by the smaller content of β-sheets in comparison to the results for phosphate buffer solutions. Moreover, it was possible to determine the dissociation constant in the presence of HSA for both unlabeled hot spots (**5**) and (**6**), as 14.4 µM (**5**) and 246 nM (**6**), respectively, using the microscale thermophoresis (MST) assay. The ability to determine both dissociation constants proves the lack of aggregation by peptides **5** and **6** in the presence of HSA. The lack of aggregation by HSA complexes with insulin and its hot spot fragments H-LYQLENY-OH (**5**) and H-VEALYL-OH (**6**) was confirmed in studies with Congo Red (CR), Thioflavin T (ThT), and in microscopic tests. 

All peptide-probe conjugates **1**, **2**, and **3** together with their unlabeled analogues **4**, **5**, **6** were able to interact with HSA. The inhibition of the aggregation process for peptides forming amyloid structures suggests that HSA may be an internal stabilizing factor of insulin and its fragments, and could therefore offer a natural, internal inhibitor of the process of their aggregation. Therefore, HSA has the potential to be used as an effective inhibitory system for amyloidogenic cores of peptides in the bloodstream. This study provides the first indication that HSA could be used as a drug delivery system for peptidic analogues of hot spots of insulin, to inhibit the aggregation of this hormone. Studies on the preparation of HSA complexes with peptide inhibitors of insulin aggregation and on the use of HSA as a transporter are ongoing.

## Figures and Tables

**Table 1:** Best fit parameters and average lifetimes <*τ*> for Py-Ins fluorescence decays in buffer, TX-100 and HSA solutions, according to bi-exponential kinetics.

	*τ_1_* [ns]	*A_1_*	*τ_2_* [ns]	*A_2_*	<*τ*> [ns]
Py-Ins/buffer (non-filtered)	17.5	0.5	98.4	0.5	86.6
Py-Ins/buffer (filtered)	20.1	0.2	98.8	0.8	95.2
Py-Ins/TX-100 (non-filtered)	17.7	0.2	152.7	0.8	147.9
Py-Ins/TX-100 (filtered)	18.2	0.1	162.0	0.9	161.2
Py-Ins/HSA (non-filtered)	19.1	0.4	143.8	0.6	133.6
Py-Ins/HSA (filtered)	19.4	0.2	150.4	0.8	145.6

**Table 2:** Lifetimes of Py-LYQLENY (**2**) and Py-VEALYL (**3**) fluorescence decay in PBS and in the presence of HSA according to mono- and bi-exponential kinetics, respectively.

Conjugate	*τ*_PBS_ [ns]	*τ*_HSA_ [ns]
Py-LYQLENY (**2**)	38.3	186.2
Py-VEALYL (**3**)	43.9	153.9

## Supplementary Materials

The following are available online at https://www.mdpi.com/2218-273X/10/10/1366/s1, Figure S1: UV-Vis spectra recorded on the first, second, and third days of incubation of HSA-LYQLENY complex in the presence of Congo Red, Figure S2: UV-Vis spectra recorded on the first, second, and third days of incubation of HSA-VEALYL complex in the presence of Congo Red, Figure S3: The intensity of fluorescence spectra recorded on the first, second, and third days of incubation of HSA-LYQLENY complex in the presence of Thioflavin T, Figure S4: The intensity of fluorescence spectra recorded on the first, second, and third days of incubation of HSA-VEALYL complex in the presence of Thioflavin T, Figure S5: Fluorescence spectra of PBA in the function of HSA centration. [PBA] = 15 µM, λ_exc_ = 337 nm. Insert: Intensity of fluorescence recorded at 376 nm as a HSA concentration, Figure S6: Time profiles of PBA [15 µM] fluorescence decay in buffer and in the presence of HSA; Figure S7: Panel I: Normalized absorption spectra of Py-LYQLENY (a) and Py-VEALYL (b) in different solvents. Panel II: Normalized emission spectra of Py-LYQLENY (a) and Py-VEALYL (b) in different solvents, λ_exc_ = 342 nm, Figure S8: Fluorescence spectra of Py-LYQLENY in PBS solutions recorded for the given probe concentration (a), normalized spectra for minimum and maximum concentrations (b), λ_exc_ = 337 nm, Figure S9: Dependence of fluorescence intensity of Py-LYQLENY (2), registered at λ = 376 nm, as a function of concentration, λ_exc_ = 337 nm, Figure S10: Transient absorption spectrum of pulse irradiated (17 ns, dose 55 Gy) aqueous solution of Py-LYQLENY (20 µM) containing 0.2 M t-BuOH. Nitrogen saturated, Table S1: Best fit parameters and average lifetimes <*τ*> for PBA fluorescence decays recorded in buffer with and without HSA.
