# Supplementary material for: Human Serum Albumin Binds Native Insulin and Aggregable Insulin Fragments and Inhibits Their Aggregation
Source: Biomolecules. 2020 Sep 25;10(10):1366. doi: 10.3390/biom10101366 (PMC7601681; doi:10.3390/biom10101366)
Supplement: Supplementary file 1 [file biomolecules-10-01366-s001.pdf]

Human serum albumin binds native insulin and aggregable insulin fragments and inhibits their aggregation

Joanna Wasko [1], Marian Wolszczak [2], Zbigniew J. Kaminski [1], Malgorzata Steblecka [1] and Beata Kolesinska [1,*]

[1] Faculty of Chemistry, Institute of Organic Chemistry, Lodz University of Technology, Zeromskiego 116, 90-924 Lodz, Poland; joanna.wasko@dokt.p.lodz.pl, zbigniew.kaminski@p.lodz.pl, steblecka.m@gmail.com

[2] Faculty of Chemistry, Institute of Applied Radiation Chemistry, Lodz University of Technology, Wroblewskiego 15, 93-590 Lodz, Poland; marian.wolszczak@p.lodz.pl

* beata.kolesinska@p.lodz.pl; Tel.: 48-42-631-32-61

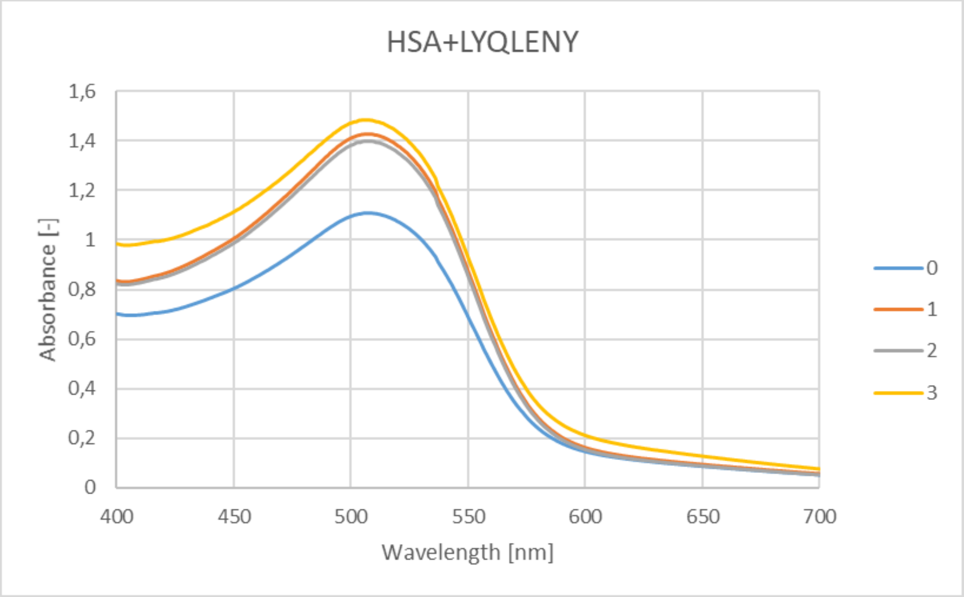

Fig. S1. UV-Vis spectra recorded on the first, second, and third days of incubation of HSA-LYQLENY complex in the presence of Congo Red.

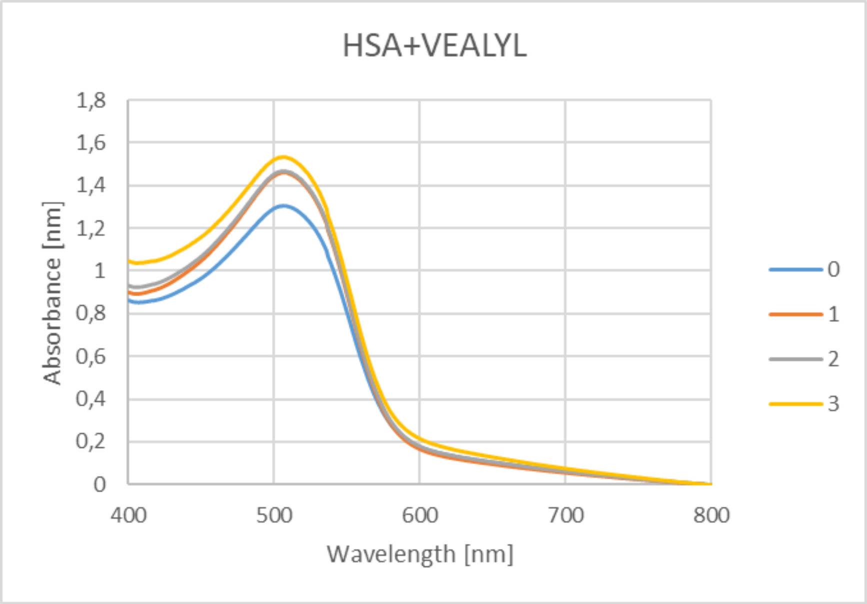

Fig. S2. UV-Vis spectra recorded on the first, second, and third days of incubation of HSA-VEALYL complex in the presence of Congo Red.

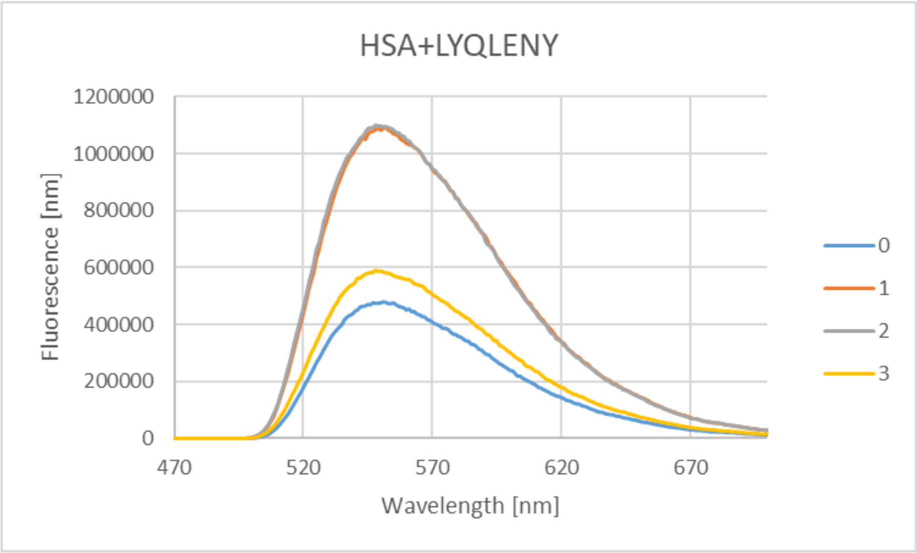

Fig. S3. The intensity of fluorescence spectra recorded on the first, second, and third days of incubation of HSA-LYQLENY complex in the presence of Thioflavin T.

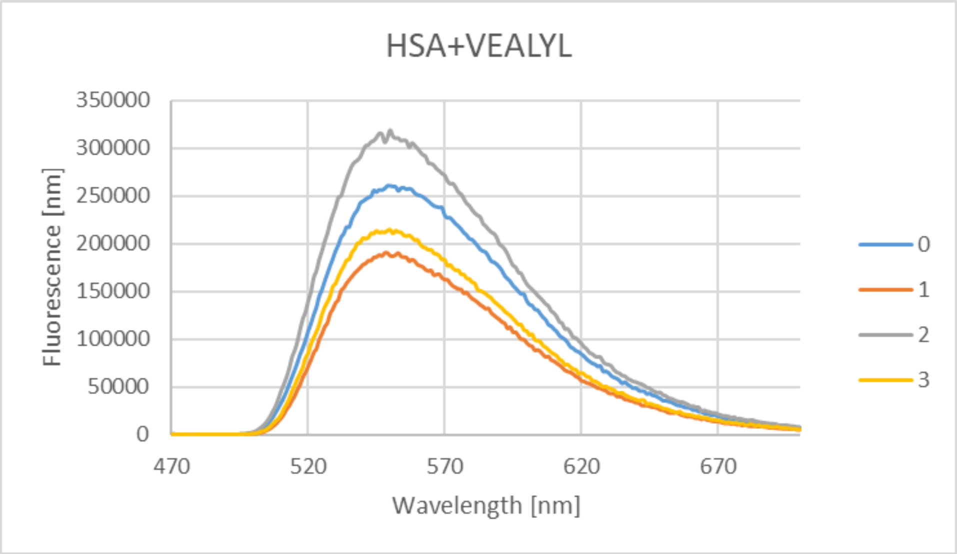

Fig. S4. The intensity of fluorescence spectra recorded on the first, second, and third days of incubation of HSA-VEALYL complex in the presence of Thioflavin T.

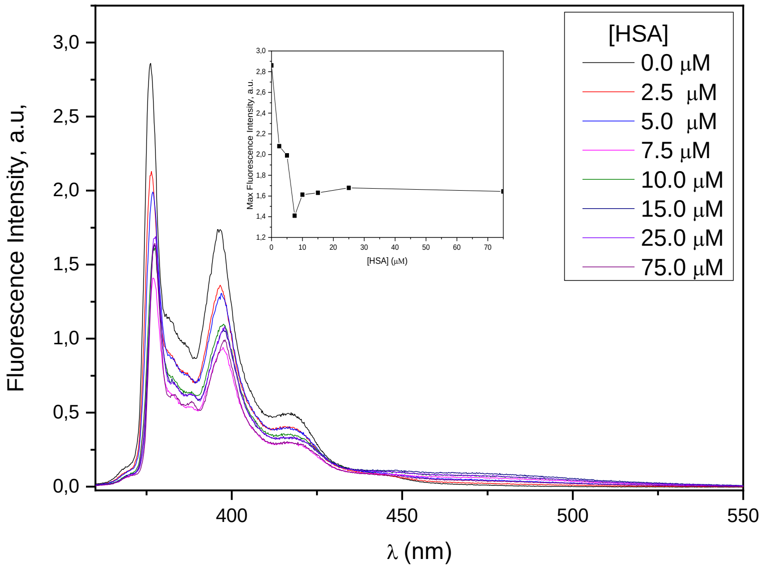

Fig. S5. Fluorescence spectra of PBA in the function of HSA centration. [PBA]=15 μM, λ_{exc}=337 nm. Insert: Intensity of fluorescence recorded at 376 nm as a HSA concentration.

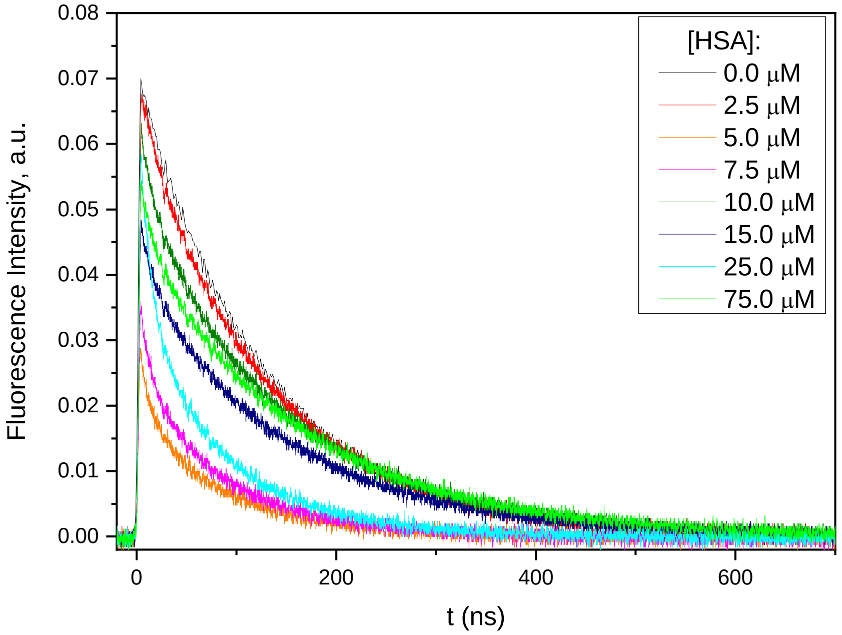

Fig. S6. Time profiles of PBA [15µM] fluorescence decay in buffer and in the presence of HSA.

Table S1. Best fit parameters and average lifetimes <τ> for PBA fluorescence decays recorded in buffer with and without HSA.

[HSA] (µM)	Time of PBA fluorescence decay (ns)	
	τ_1	τ_2
0.0	126	X
2.5	126	
5.0	130	
7.5	18	147
10.0	20	150
15.0	19	154
25.0	18	157
75.0	16	163

The absorbances of Py-LYQLENY and Py-VEALYL were determined in 10 mM standard PBS solution, ethanol, methanol, and 10 mM TX-100. The spectra were normalized to a maximum of $S_0 \rightarrow S_2$ band (Figure 4, panel I). A specific oscillation structure characteristic for pyrene derivatives was found, with the maximum absorbance at around 340 nm. Narrow absorption bands in ethanol and methanol indicated that the solubility of the probes was good in these media. For both labeled peptides **2** and **3** in methanol, a shift of 2 nm towards shorter wavelengths was observed in comparison to the spectra in the buffer, while the spectrum in Triton TX-100 was shifted by 4 nm to red (batochromic shift). This may have been the result of locating the pyrene probe in a hydrophobic microenvironment [42]. In the buffer solution, the band with the highest intensity was significantly broadened, which could indicate the presence of aggregates affecting the spectral properties of the pyrene-labeled peptides.

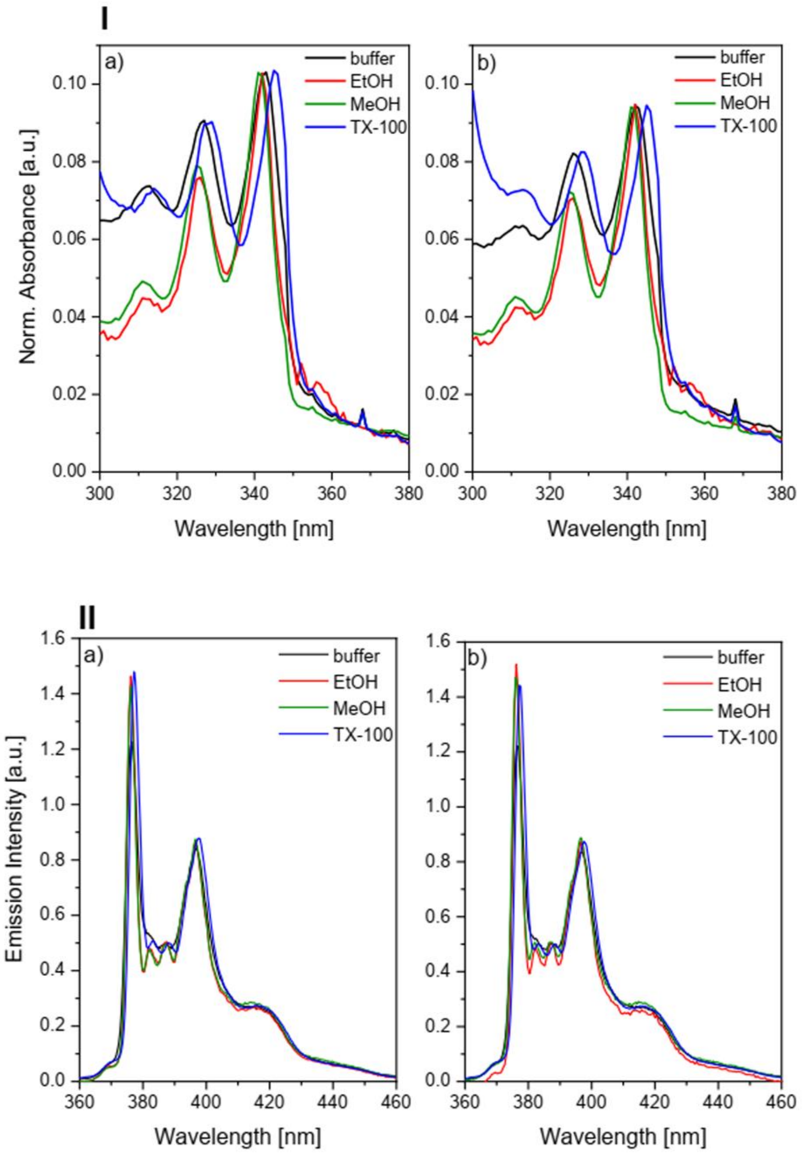

Fig. S7. Panel I: Normalized absorption spectra of Py-LYQLENY (a) and Py-VEALYL (b) in different solvents. Panel II: Normalized emission spectra of Py-LYQLENY (a) and Py-VEALYL (b) in different solvents, λ_{exc} = 342 nm.

We also investigated the solubility of Py-LYQLENY conjugate (**2**) in dioxane. The sample dissolved relatively well. This allowed the molar coefficient absorbance to be determined, using the gravimetric method. It was found to be equal to 35 000 $dm^3\ mol^{-1}\ cm^{-1}$. The solubility of conjugate **2** in the buffer solution was lower, but after shaking the samples it was possible to determine the molar absorbance coefficient of Py-LYQLENY as 17 000 $dm^3\ mol^{-1}\ cm^{-1}$. The addition of a small amount of concentrated Py-LYQLENY stock solution in dioxane to the buffer solution enabled the molar absorbance coefficient to be found. For a concentration of Py-LYQLENY below 1 μM, as measured in quartz cell of 10 cm optical path it was 29 800 $dm^3\ mol^{-1}\ cm^{-1}$. These results can be explained by assuming that the pyrene derivative aggregates (mainly forming dimers) in an aqueous buffer solution. Similar changes in the molar absorption coefficient due to aggregation have been observed by Siu and Duhamel [43] for a short poly(ethylene oxide) derivative labeled with 1-pyrenemethoxide.

The results of the absorption tests were also confirmed in fluorescence studies Py-LYQLENY and Py-VEALY (Figure 4, Panel II). On emission spectra normalized to the third band, five characteristic

bands are visible, of which the first and fourth have the highest intensity and the last band is significantly distorted. The high ratio of I_1/I_3 intensities confirms the polar environment of the pyrene label, based on the pyrene derivatives polarity scale [42].

The lower degree of vibrational coupling by the pyrene probe with water than with alcohol may be shown by the lack of a subtle fluorescence structure for the probe in PBS. In order to investigate the possible aggregation of the fluorescence labeled insulin hot spots in the buffer solution, spectrophotometric titration was performed for Py-LYQLENY (**2**) in a range of concentration from 2.9 to 56.4 μM. Titration was carried out until there were no significant changes in fluorescence intensity. The fluorescence spectra were recorded for each concentration (Figure 5a). After normalization of the intensity of the spectra for two extreme concentrations, 2.9 μM and 56.4 μM (Figure 5b), a significant difference was observed in the 400–520 nm band. The intensity of this band in the case of higher concentrations may result from the formation of excimers between the aggregated molecules.

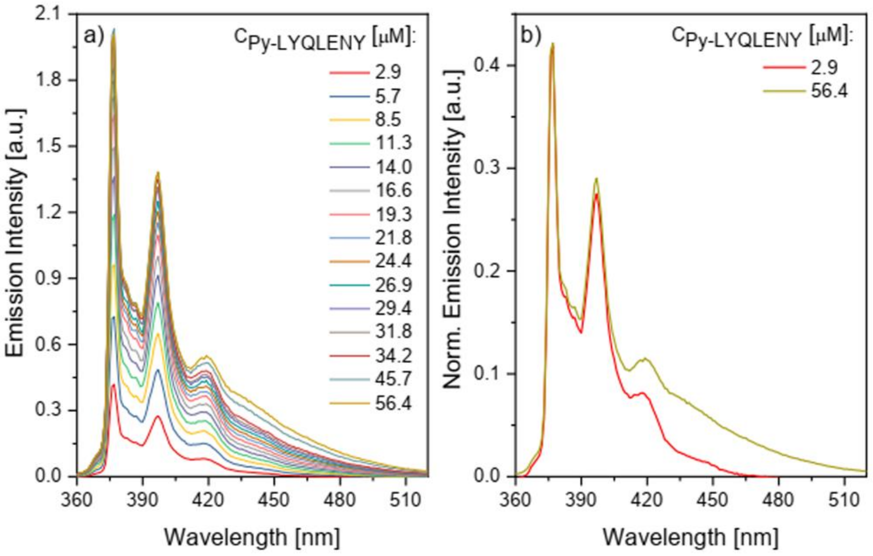

Fig. S8. Fluorescence spectra of Py-LYQLENY in PBS solutions recorded for the given probe concentration (a), normalized spectra for minimum and maximum concentrations (b), λ_{exc} = 337 nm.

Figure 6 illustrates the dependence of the fluorescence intensity registered at 376 nm on the concentration of Py-LYQLENY (2) in PBS. Initially, increasing the concentration of Py-LYQLENY induces a rise in the fluorescence intensity. However, when the concentration of the probe exceeds 30 μM, the emission intensity does not increase any further.

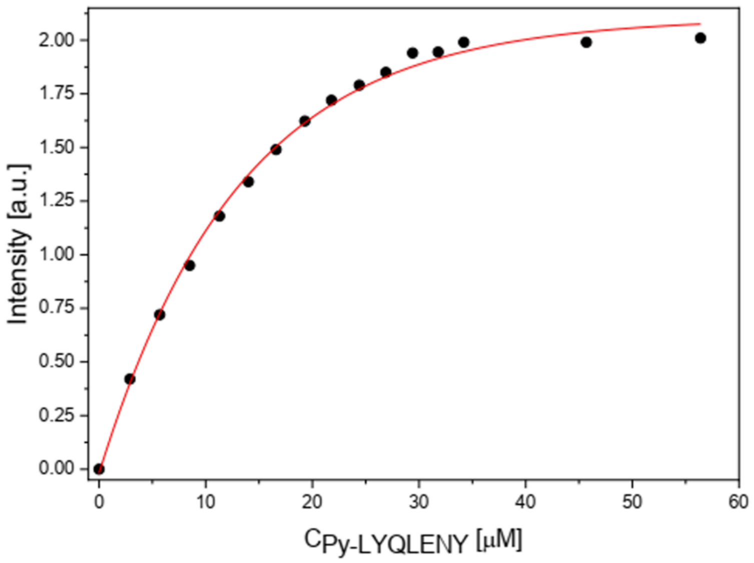

Fig. S9. Dependence of fluorescence intensity of Py-LYQLENY (2), registered at λ=376 nm, as a function of concentration, λ_{exc} = 337 nm.

The observed plateau may result from the aggregation of Py-LYQLENY and the formation of excimers. The fluorescence of the probe within the aggregates is immediately quenched (static quenching), so increasing the concentration does not induce any further increase in the intensity of emission.

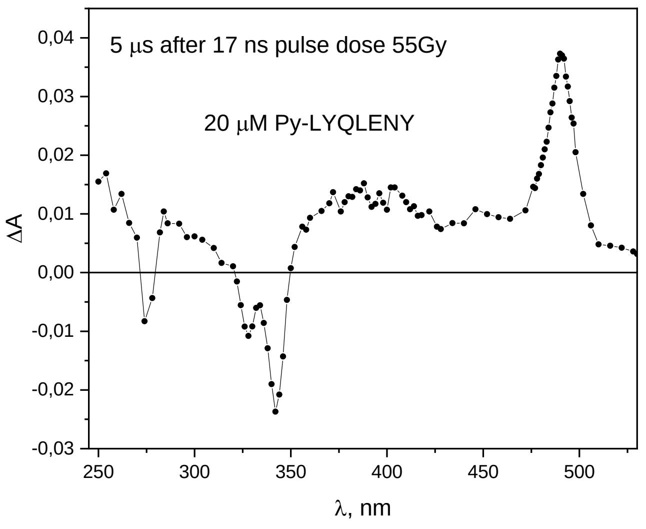

Fig. S10. Transient absorption spectrum of pulse irradiated (17 ns, dose 55 Gy) aqueous solution of Py-LYQLENY (20 µM) containing 0.2 M t-BuOH. Nitrogen saturated.
